# Dynamic microRNA Responses Contribute to Phenotypic Plasticity and Stress Memory in Invasive Species

**DOI:** 10.1111/mec.70160

**Published:** 2025-10-22

**Authors:** Zhenyong Du

**Affiliations:** ^1^ Department of Integrative Biology University of Wisconsin Madison Wisconsin USA

**Keywords:** biological invasions, climate change, environmental adaptation, epigenetic regulation, microRNA (miRNA), phenotypic plasticity, stress memory

Phenotypic plasticity enables organisms to rapidly adjust to environmental challenges, yet the molecular mechanisms underlying these plastic responses remain incompletely understood, particularly in invasive species. In a recent publication in Molecular Ecology, Yan et al. reveal how dynamic microRNA (miRNA)‐mediated gene regulation facilitates rapid and reversible phenotypic adjustments in the invasive ascidian *Ciona robusta* in response to recurrent salinity stress. Using integrated transcriptomic and miRNA profiling, the authors identify highly context‐dependent miRNA responses that not only regulate osmotic stress pathways but also confer a molecular ‘stress memory’, enhancing subsequent responses to repeated environmental challenges. These findings advance our understanding of how epigenetic mechanisms, such as miRNA regulation, contribute to plasticity‐driven resilience during invasions and environmental fluctuations. Integrating these insights with experimental evolution approaches will be crucial for unravelling the long‐term evolutionary implications of short‐term plastic responses and more accurately predicting organismal responses under global climate change.

Phenotypic plasticity is the ability of an organism to produce different phenotypes from the same genotype under varying environmental conditions. It is increasingly recognised as a critical factor determining the survival and persistence of species confronted by rapid ecological changes (Pigliucci 2005; Sommer 2020). Biological invasions and global climate change subject organisms to rapid and recurrent environmental challenges (Bernatchez et al. 2024; Chown et al. 2015). Invasive species, in particular, often experience harsh and fluctuating conditions during transport, introduction, and establishment in new habitats. Biological invasions offer natural experimental frameworks for studying phenotypic plasticity, as invasive species must rapidly adapt to novel environmental conditions encountered during the invasion process (Lee 2002). Although genetic adaptation is a fundamental driver of evolutionary change, epigenetic mechanisms such as microRNA (miRNA)‐mediated gene regulation offer rapid and reversible avenues for organisms to cope with fluctuating environmental challenges (Biggar and Storey 2015). Using the invasive ascidian *Ciona robusta* as a model system (Figure 1), Yan et al. (2025) provide compelling evidence that dynamic miRNA regulation contributes to rapid phenotypic plasticity and stress memory during biological invasions.

**FIGURE 1:**
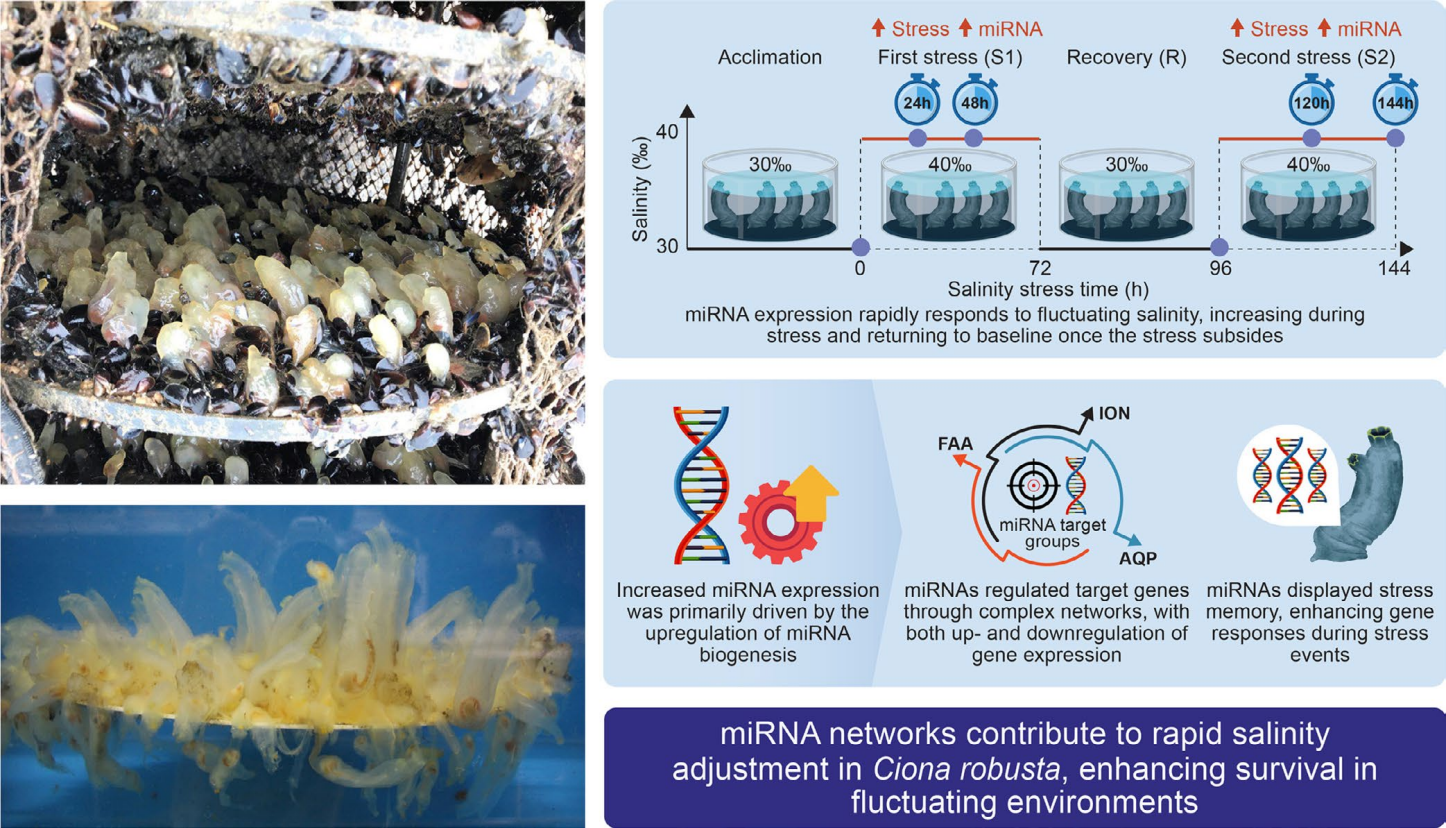
Schematic diagram summarising the main findings of Yan et al. (2025). Left: *Ciona robusta* attached to a plate in a bivalve rearing cage (top), and environmental challenge experiments conducted on collected individuals in an aquarium (bottom). Photo credit: Zhan Lab. Right: the schematic illustrates the experimental design simulating repeated salinity challenges with an intermediate recovery phase following acclimation. miRNA expression rapidly increased during each stress phase and largely returned to baseline upon recovery. Distinct sets of miRNAs were differentially expressed at each stage, reflecting stage‐specific regulatory dynamics. Elevated miRNA abundance was primarily driven by upregulation of biogenesis genes. miRNAs regulated a diverse set of target genes through both up‐ and down‐regulation, including those involved in ion transport (ION), free amino acid metabolism (FAA), and aquaporin‐mediated water transport (AQP). Notably, some miRNAs and their targets exhibited stress memory, with enhanced responses during the second stress exposure. These findings emphasise the role of miRNA‐mediated regulation in phenotypic plasticity and environmental resilience during biological invasions. Created by Zhenyong Du.

The study by Yan et al. (2025) focuses on recurrent salinity stress, a common environmental challenge faced by marine invasive species during their transport and colonisation phases (Lee et al. 2022). Using integrated approaches combining transcriptomic analyses and miRNA profiling, the authors identify rapid, reversible, and highly context‐dependent changes in miRNA expression in response to salinity fluctuations. Crucially, the observed miRNA‐mediated regulatory networks not only fine‐tune gene expression involved in osmotic regulation but also exhibit a form of molecular ‘stress memory’, where prior exposure to stress enhances the organism's subsequent response.

A central finding of this study is the demonstration of the dynamic nature of miRNA responses. Yan et al. illustrate that miRNA expression shifts rapidly in response to initial stress and then largely returns to baseline after recovery, emphasising the reversible nature of epigenetic regulation (Figure 1). Furthermore, distinct miRNA profiles emerged during different phases of stress and recovery, suggesting precise temporal control and specificity in miRNA regulatory roles. The study also sheds light on the underlying mechanism driving these dynamic changes, revealing that miRNA abundance during stress primarily increases due to enhanced transcriptional and biogenesis pathways rather than reduced miRNA degradation.

Notably, Yan et al. uncovered regulatory interactions in which miRNAs modulate gene expression networks through both repressive and unexpected up‐regulatory effects. Such sophisticated control likely enables rapid adjustments to physiological processes, including the modulation of free amino acid metabolism and ion transport, which are crucial for maintaining osmotic homeostasis (Figure 1). These findings provide a nuanced understanding of how miRNAs collectively steer organismal responses to environmental challenges, emphasising their crucial roles in fine‐tuning phenotypic plasticity.

In particular, the authors demonstrate miRNA‐mediated stress memory, in which previous exposure to salinity stress primes *C. robusta* for more effective responses to subsequent similar stresses (Figure 1). This molecular ‘memory’ was evidenced by enhanced induction of specific miRNAs and their target genes during recurrent stresses. Stress memory, observed here at the miRNA level, echoes physiological and transcriptional stress memory phenomena previously described in related studies (Li et al. 2024). This reinforces the hypothesis that ‘learning’ from past environmental encounters may be an adaptive strategy broadly employed across multiple regulatory layers.

Integrating these findings into a broader ecological and evolutionary context, the work by Yan et al. advances our understanding of epigenetic contributions to invasion success. Epigenetic plasticity, particularly through miRNA regulation, may serve as a crucial mechanism for rapid acclimation in invasive species with limited genetic diversity, acting as a buffer against sudden environmental shifts (Estoup et al. 2016). This concept aligns well with recent opinions emphasising the importance of epigenetic mechanisms in enabling rapid adaptation and resilience in response to global climate change scenarios (Bernatchez et al. 2024).

Furthermore, the multidimensional nature of plastic responses, encompassing changes in gene expression, alternative splicing, polyadenylation, and miRNA regulation (Figure 2), emphasises the complexity of molecular adjustments organisms employ to cope with stress (Pu et al. 2019; Huang et al. 2023). Each regulatory layer appears to target distinct gene sets and functional pathways (Huang et al. 2023), suggesting that coordination among different molecular mechanisms is necessary for successful environmental adaptation.

**FIGURE 2:**
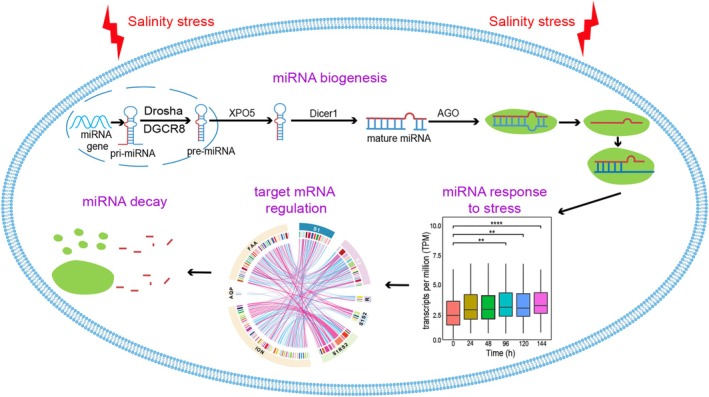
Overview of biological processes involved in miRNA‐mediated gene regulation in *Ciona robusta* in response to recurrent environmental challenges (Yan et al. 2025). The schematic outlines the key steps of miRNA biogenesis, environmental stress‐induced miRNA responses, subsequent miRNA regulation of target mRNAs, and the eventual decay of miRNAs. Created by Weijie Yan, Xuena Huang and Aibin Zhan.

The insights gained from Yan et al. suggest exciting avenues for future research, particularly in exploring how transient epigenetic responses, such as miRNA‐mediated plasticity, could influence long‐term genetic adaptation. Evolve and Resequence (E&R) experiments, which track genomic and epigenomic evolution in real‐time across multiple generations, offer a promising approach for investigating these evolutionary trajectories (Bernatchez et al. 2024; Brennan et al. 2022; Stern et al. 2022). Such experiments could illuminate whether initial miRNA‐mediated adjustments eventually lead to stable transgenerational transmission or genetic changes, thereby transforming temporary stress responses into heritable adaptations.

Moreover, understanding the potential for cross‐generational transmission of stress memory could provide deeper insights into the evolutionary implications of epigenetic plasticity. Investigating whether stress‐induced miRNA profiles can be transmitted to subsequent generations is a key area for future work, as this would illuminate additional dimensions of adaptive potential in invasive species. Further research into the underlying mechanisms facilitating such transgenerational inheritance is also warranted. Additionally, studies are needed to determine the extent to which miRNA‐mediated plastic responses observed in *C. robusta* are conserved across diverse taxa and environmental contexts.

In conclusion, Yan et al. (2025) have demonstrated that dynamic and reversible miRNA‐mediated gene regulation is a potent mechanism contributing to rapid phenotypic plasticity and stress memory in invasive species. By elucidating these molecular responses, the study provides valuable insights into how invasive organisms navigate environmental challenges. Understanding these mechanisms not only deepens our comprehension of biological invasions but also provides vital insights into how species adapt to accelerating environmental change. Future research integrating molecular plasticity studies with evolutionary experiments holds promise for unravelling the interaction between short‐term responses and long‐term adaptation. Equally important, experimental validation of predicted miRNA–target interactions, using methods such as luciferase reporter assays or gene knockdowns, will be necessary to validate in silico findings and clarify the mechanisms underlying these regulatory networks. Ultimately, these combined efforts will enhance predictions of ecological resilience and vulnerability amid global climate change and inform future studies on the adaptive capacity of invasive species.

## Author Contributions

Zhenyong Du takes full responsibility for this article.

## Conflicts of Interest

The author declares no conflicts of interest.

## Data Availability

Data sharing not applicable to this article as no datasets were generated or analysed during the current study.
